# COVID‐19 Infection and Massive Aortic Dissection

**DOI:** 10.1002/ccr3.9659

**Published:** 2024-12-03

**Authors:** Mehrangiz Zangeneh, Yasamin Khosravaninezhad, Masoumeh Mesgarian

**Affiliations:** ^1^ Infectious Diseases Department Islamic Azad University of Medical Sciences Tehran Iran; ^2^ Health Policy Center Shiraz University of Medical Sciences Shiraz Iran

**Keywords:** aorta dissection, cardiovascular, case report, COVID‐19, viral infection

## Abstract

COVID‐19 had a significant impact on public health, including respiratory and cardiovascular complications. Because of COVID‐19 hypercoaglupathy effect, it can lead to cardiovascular complications. A 68‐year‐old Iranian male admitted to the infectious diseases department with the diagnosis of COVID‐19 infection. Despite initially stable vital signs and mild symptoms, the patient's condition rapidly deteriorated over the course of several days, with severe respiratory distress and other concerning symptoms. Further investigations revealed evidence of aortic aneurysm or dissection, which was confirmed to be a Stanford type A aortic dissection extending from the aortic root to the abdominal aorta. The available evidence points to a potential association between COVID‐19 infection and aortic dissection, and the need for continue investigations about the pathophysiological mechanisms underlying this relationship.

**Summary**

- Our case underscores the potential association between COVID‐19 infection and aortic dissection in a patient with no previous history of underlying diseases, suggesting a need for heightened vigilance and investigation into cardiovascular complications in patients with COVID‐19, which is still mysterious.

## 1 Introduction

The novel coronavirus caused the coronavirus 2019 (COVID‐19) pandemic, which was officially declared as global pandemic by the World Health Organization in 2020. The virus first reported to the World Health Organization [1] in November 2019 as emerged virus in Wuhan, China [2]. COVID‐19 had a significant impact on public health, leading to respiratory and cardiovascular complications, and has posed challenges to global socio‐economy and education [3]. The evolving trajectory of COVID‐19 has prompted extensive research on immunity and the development of vaccines to prevent and control the spread of the virus [4]. At the last report in January 2024, the total number of confirmed COVID‐19 cases worldwide was 702,206,819, and the total number of deaths was 6,972,613 [1]. These numbers show there are new cases and deaths that are reported daily. The clinical symptoms of COVID‐19 primarily are from the respiratory and cardiovascular systems, as severe pneumonia being a significant complication, especially in diabetic patients. Additionally, a higher risk of disease progression and poor prognosis are obvious in individuals with cardiovascular diseases. COVID‐19 has been associated with significant morbidity, mortality, and long‐term health effects, with more than 200 documented symptoms affecting multiple organ systems. SARS‐CoV‐2 enters the body's cells via angiotensin‐converting enzyme 2 (ACE2) receptors, causing COVID‐19–associated pneumonia, acute damage to the heart, and long‐term harm to the cardiovascular system. The cardiac issues arising from acute COVID‐19, such as myocardial injury, myocarditis, acute heart attacks, heart failure, irregular heartbeats, and blood clotting disorders, have been extensively recorded [5]. COVID‐19 has been linked to increased blood clotting tendencies, which may result in cerebrovascular conditions and complications related to the aorta [6]. The relationship between COVID‐19 and aortic dissection has been highlighted in several studies. Some cases have shown significant aortic wall thickening in COVID‐19 patients with aortic dissection, indicating inflammatory aortic pathologies [7]. Additionally, there have been reports of acute type A aortic dissection in patients with recent COVID‐19 infections, suggesting a potential association between two conditions [8]. Aortic dissection is a serious medical condition characterized by the tearing of the inner layer of the aorta, the body's primary artery, resulting in a potentially life‐threatening separation of its layers. This condition poses significant risks such as organ damage, stroke, or fatal internal bleeding. Age, particularly being over 60, male gender, certain connective tissue ailments, and activities such as heavy weightlifting also heighten the risk [9]. Prompt diagnosis and effective treatment of aortic dissection during the COVID‐19 pandemic are critical to prevent complications and reduce pressure on healthcare systems already dealing with increased demands. This case report describes a male patient with a primary diagnosis of COVID‐19 who subsequently experienced a massive aortic dissection, resulting in death.

## 2 Case History

A 68‐year‐old man from Iran was hospitalized in the infectious diseases ward on September 25, 2021, with a primary diagnosis of COVID‐19. Upon admission, the patient presented with symptoms of fever, chills, loss of smell, and loss of taste, which had persisted for 1 week. He denied any history of diabetes, hypertension, heart disease, or thyroid disorders. His medical history was notable for cataract surgery and benign prostatic hyperplasia, for which he was taking finasteride and tamsulosin. The patient also reported a previous COVID‐19 infection 1 year prior to the current admission. On admission, his vital signs were as follows: oxygen saturation 95% on room air, pulse rate (PR) 52 beats per minute, blood pressure (BP) 110/70 mmHg, and respiratory rate 20 breaths per minute. A chest computed tomography (CT) scan revealed approximately 20% diffuse ground‐glass opacities (GGO). His electrocardiography (ECG) was normal. Physical examination indicated a generally fair condition, with normal heart sounds and mild bilateral rhonchi in the lungs. All other systems and physical examinations were unremarkable. Real‐time polymerase chain reaction (PCR) confirmed the presence of SARS‐CoV‐2. The patient was treated with remdesivir, heparin, dexamethasone, and pantoprazole following admission.

Four days after admission, the patient developed a severe, productive cough, and his oxygen saturation dropped to 90% on room air, improving only slightly to 93% with a nasal cannula. During physical examination, oral thrush caused by *Candida albicans* was also detected. Lab results revealed a drop in platelet count, with a significantly elevated D‐dimer level of 900 mg/L, raising concerns. The next day, his condition worsened—his breathing became more labored, and a follow‐up chest CT showed a marked increase in lung involvement, with 50% diffuse GGO (PR: 87, oxygen saturation: 84% on room air). Given the elevated D‐dimer levels and worsening symptoms, a lung CT angiography was performed to rule out a pulmonary embolism, which came back negative. However, his condition took a more concerning turn when he began coughing up bloody sputum, feeling dizzy, and reporting extreme fatigue (PR: 85, oxygen saturation: 80% on room air). The following day, he suddenly experienced severe chest pain, which he described as a heavy pressure on his chest, along with worsening shortness of breath.

## 3 Method

**FIGURE 1:**
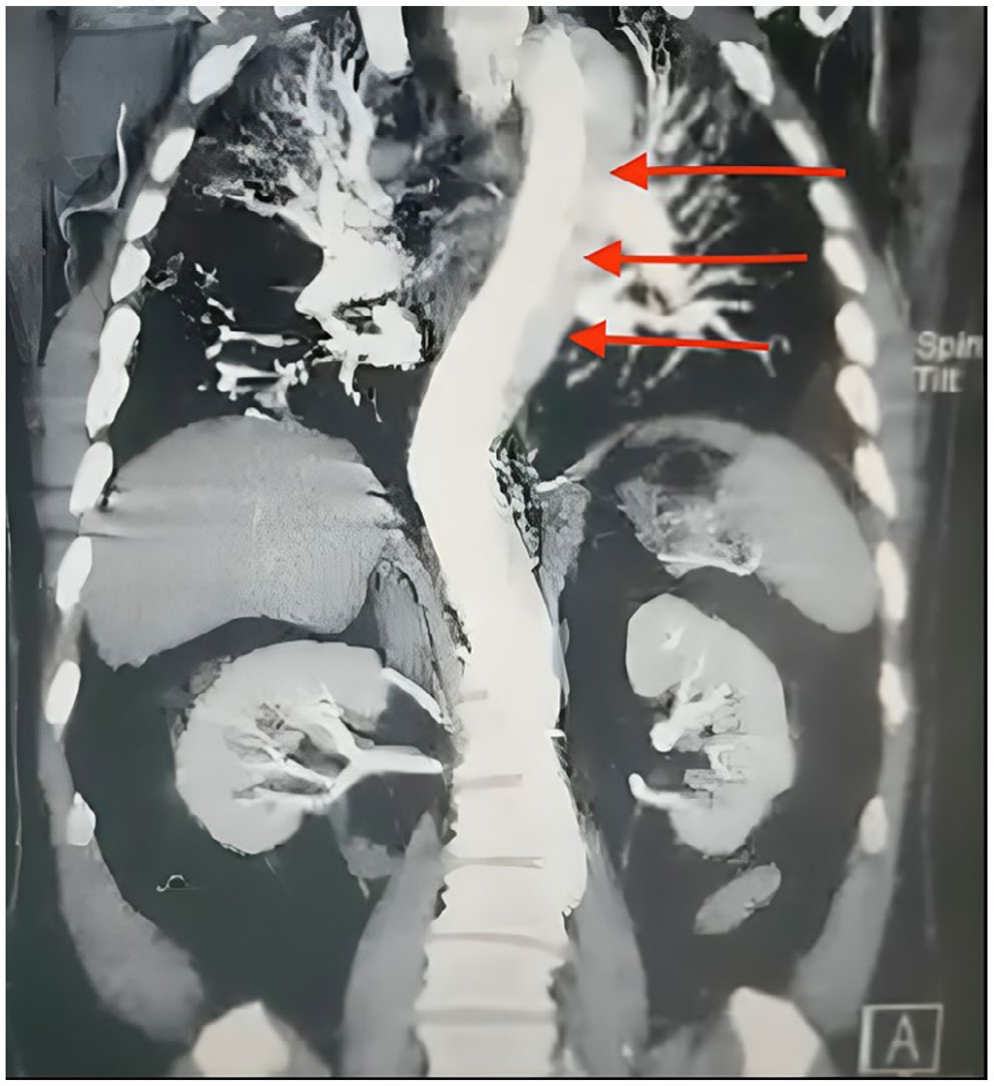
2D CT angiography of the chest and abdomen showing aortic dissection (red arrows). The dissection involves Stanford type A, where the intimal layer of the aorta separates, allowing blood to flow between the layers of the vessel wall. This dissection extends from the aortic root through the ascending aorta and into the abdominal aorta.

A bilateral carotid and vertebral artery color Doppler sonography was performed, and the results were normal. However, upon consultation with a cardiologist, echocardiography revealed a concerning finding—either an aortic aneurysm or dissection, with the heart's ejection fraction measuring at 55%. A chest X‐ray added to the concerns, showing diffuse reticular consolidation and mediastinal widening. As the patient's condition worsened and became critical, a neurologist was consulted. A brain CT scan revealed mild cerebral atrophy, likely due to aging, but nothing significant related to the current illness. Given the urgency of the situation, an emergency consultation with a cardiac surgeon was called for. A second CT angiography of the chest and abdomen confirmed the presence of a massive aortic dissection. The dissection originated from the aortic root and extended through the ascending aorta, aortic arch, descending thoracic aorta, and abdominal aorta, reaching down to the level of the superior mesenteric artery (Stanford type A) (Figure 1). Furthermore, the celiac artery was found to arise from the false lumen, and there was aneurysmal dilation of the ascending aorta, which required high‐risk vascular surgery.

## 4 Results

Despite preparations for cardiac surgery, the patient passed away the following day (details provided in Table 1).

**TABLE 1:** Case summary.

Signs	Days					
	Day 1	Day 4	Day 5	Day 6	Day 7	Day 8
CC	Mild dyspnea	Dyspnea worsening + oral fungus	Bloody sputum	Severe dyspnea	Sudden chest pain	Severe illness
VS	O_2_ saturation: 95% PR: 52 BP: 110/70 RR: 20	O_2_ saturation: 84% PR: 87	O_2_ saturation: 85% PR: 85	O_2_ saturation: 84%	O_2_ saturation: 83%	BP: 110/65 PR: 77
CT scan	20% GGO	50% GGO	—	—	—	—
CT angiography	—	No evidence of lung embolism	—	—	Dissection of the aorta	—
Laboratory	—	D‐dimer: 900 mg/L + platelets: 153,000/ mm^3^	D‐dimer: 1589 mg/L BUN: 39 mg/dL Cr: 1.3 mg/dL procalcitonin: 0.337 ng/dL	—	FBS: 261 mg/dL BUN: 36 mg/dL LDH: 502 IU/L	Ferritin 730 ng/mL
Treatment and results	Remdesivir, heparin, dexamethasone, and pantoprazole	Adding of ceftriaxone and azithromycin and fluconazole	Antibiotic change to meropenem	Adding of methyl‐prednisolone and tocilizumab	Methyl‐prednisolone and tocilizumab discontinued	Expired during surgery preparation

Abbreviations: BP, blood pressure; BUN, blood urea nitrogen; CC, chief complain; Cr, creatinine; FBS, fasting blood sugar; GGO, ground glass opacity; IU/L, international unit per liter; mg/dL, milligrams per deciliter; mg/L, milligrams per liter; mm, millimeter; ng/dL, nanograms per deciliter; ng/mL, nanograms per milliliter; PR, pulse rate; RR, respiratory rate; VS, vital signs.

## 5 Discussion

The case involves a 68‐year‐old Iranian male admitted to the infectious diseases department with a primary diagnosis of COVID‐19. Despite initially stable vital signs and mild symptoms, the patient's condition rapidly deteriorated over the course of several days, with severe respiratory distress and other concerning symptoms. Further investigations revealed evidence of aortic aneurysm or dissection, which was confirmed to be a Stanford type A aortic dissection extending from the aortic root to the abdominal aorta. At the admission time, the worldwide dominant COVID‐19 variant was Delta. Despite preparations for surgery, the patient unfortunately passed away. This case emphasize the complexity and potential complications associated with COVID‐19 and highlighting the importance of complete evaluation and interdisciplinary management in critically ill patients.

Aortic dissection in the context of COVID‐19 point to attention to potential associations between COVID‐19–induced inflammatory endothelial damage and vessel wall pathologies, including aortic dissection [10]. COVID‐19 seems to correlate with a heightened risk and seriousness of acute type A aortic dissection, an urgent and life‐threatening condition necessitating immediate surgical intervention. Various case reports and studies have documented COVID‐19 patients experiencing severe aortic dissections. Even with timely diagnosis and treatment, mortality rates have been notably elevated compared to non–COVID‐19 counterparts [7, 11]. Additionally, there have been cases of acute type A aortic dissections successfully managed after delaying surgery in patients with recent COVID‐19 infections [8]. The presence of COVID‐19 has been linked to a higher occurrence of acute pulmonary embolism subsequent to acute type A aortic dissection, especially in individuals with coagulation irregularities [12]. While the exact causal relationship between COVID‐19 and aortic dissection is still being explored, there is growing recognition of the potential association between the two conditions [13]. The literature suggests that COVID‐19 may contribute to aortic dissection through mechanisms such as aortitis and endothelial cell damage induced by inflammatory responses [14]. The suggested mechanisms linking COVID‐19 and aortic dissection are as follows: (1) Direct injury to vascular endothelium: SARS‐CoV‐2 can directly harm the vascular endothelium, which may weaken the aortic wall. (2) Hyperinflammatory response and cytokine storm: COVID‐19 can induce a hyperinflammatory state and cytokine storm, potentially leading to plaque rupture and subsequent dissection. (3) Medication effects: Certain hypertension medications like ACE inhibitors and angiotensin receptor blockers (ARBs) could upregulate the ACE2 receptor, which the SARS‐CoV‐2 virus utilizes to enter cells. This upregulation might increase vascular susceptibility, thereby contributing to aortic vulnerability [15]. The findings from the search results also highlight that COVID‐19 has resulted in delays in diagnosing and treating acute aortic dissections. This delay can be attributed to strained healthcare resources and patients' reluctance to seek medical attention. Unfortunately, postponing treatment can exacerbate surgical challenges and worsen patient outcomes [11]. The COVID‐19 pandemic had a noticeable impact on healthcare delivery, especially when it came to managing urgent cardiovascular conditions like type A aortic dissection. Hospitals had to reorganize their departments, and strict protocols were necessary to keep surgeries running safely. At the same time, the pandemic caused a sharp drop in routine diagnostic and therapeutic care for hypertension—a major risk factor for aortic dissection. This lack of regular care likely led to delayed diagnoses and suboptimal blood pressure management in many patients, increasing the risk of serious cardiovascular complications [16]. This case highlights how important it is to address these issues as we continue to understand the broader impact of COVID‐19 on cardiovascular outcomes. In summary, the evidence indicates that COVID‐19 significantly increases the risk of acute aortic dissection, leading to higher severity and mortality rates compared to cases not related to COVID‐19. Swift diagnosis and surgical intervention remain essential for these patients, even amidst the additional challenges presented by the pandemic. To conclude, the correlation between COVID‐19 and aortic dissection is an ongoing subject of research and clinical focus. Current evidence suggests a potential link between the two, underscoring the importance of further exploration into the underlying pathophysiological mechanisms.

## Author Contributions

**Mehrangiz Zangeneh:** conceptualization, supervision, validation, writing – review and editing. **Yasamin Khosravaninezhad:** conceptualization, writing – original draft, writing – review and editing. **Masoumeh Mesgarian:** conceptualization, visualization, writing – review and editing.

## Ethics Statement

Written informed consent was obtained from patient to publish this report in accordance with the journal's patient consent policy.

## Consent

Not applicable, as all patient data has been fully anonymized.

## Conflicts of Interest

The authors declare no conflicts of interest.

## Data Availability

Data are available to be respectfully shared as the editor may request.

## References

[1] WHO , “Number of COVID‐19 Cases Reported to WHO,” https://data.who.int/dashboards/covid19/cases?n=c 2024.

[2] M. Ciotti , M. Ciccozzi , A. Terrinoni , W. C. Jiang , C. B. Wang , and S. Bernardini , “The COVID‐19 Pandemic,” Critical Reviews in Clinical Laboratory Sciences 57 (2020): 365–388, 10.1080/10408363.2020.1783198. 32645276

[3] B. Debata , P. Patnaik , and A. Mishra , “COVID‐19 Pandemic! It's Impact on People, Economy, and Environment,” Journal of Public Affairs 20 (2020): e2372, 10.1002/pa.2372.

[4] A. Chouhdari , Y. Khosravani‐Nezhad , T. Tarjoman , and M. Zangeneh , “Basic Characteristics of Patients With COVID‐19 After SARS‐CoV‐2 Vaccine: One Cross‐Sectional Study in Iran,” Vacres 9 (2022): 34–38, 10.52547/vacres.8.2.88.

[5] B. Long , W. J. Brady , A. Koyfman , and M. Gottlieb , “Cardiovascular Complications in COVID‐19,” American Journal of Emergency Medicine 38 (2020): 1504–1507, 10.1016/j.ajem.2020.04.048. 32317203 PMC7165109

[6] M. A. Ellul , L. Benjamin , B. Singh , et al., “Neurological Associations of COVID‐19,” Lancet Neurology 19 (2020): 767–783, 10.1016/S1474-4422(20)30221-0. 32622375 PMC7332267

[7] R. Irilouzadian , H. Salehi Omran , and T. Alirezaei , “Fatal Association of COVID‐19 and Acute Type A Aortic Dissection,” Clinical Case Reports 10 (2022): e05617, 10.1002/ccr3.5617. 35356160 PMC8939039

[8] M. Robu , D. R. Marian , R. Vasile , et al., “Delayed Surgical Management of Acute Type A Aortic Dissection in a Patient With Recent COVID‐19 Infection and Post‐COVID‐19 Bronchopneumonia—Case Report and Review of Literature,” Medicina 58 (2022): 1357. 36295518 10.3390/medicina58101357 PMC9609154

[9] Clinic M , “Aortic Dissection,” https://www.mayoclinic.org/diseases‐conditions/aortic‐dissection/symptoms‐causes/syc‐20369496 2021.

[10] V. Silvestri and G. E. Recchia , “Aortic Pathology During COVID‐19 Pandemics. Clinical Reports in Literature and Open Questions on the Two Co‐Occurring Conditions,” Annals of Vascular Surgery 75 (2021): 109–119, 10.1016/j.avsg.2021.02.037. 33823253 PMC8018903

[11] A. Lyon , Z. Gunga , L. Niclauss , V. Rancati , and P. Tozzi , “Case Report: Are We Witnessing an Increase of Chronic Ascending Aortic Dissection as a Collateral Effect to the COVID‐19 Pandemic?,” Frontiers in Cardiovascular Medicine 8 (2021): 645135. 33996941 10.3389/fcvm.2021.645135 PMC8113377

[12] D. Volvovitch , E. Ram , H. Cohen , A. Kogan , L. Sternik , and E. Raanani , “Acute Pulmonary Embolism Following Acute Type A Aortic Dissection in a Patient With COVID‐19,” Journal of Cardiac Surgery 36 (2021): 1566–1568, 10.1111/jocs.15389. 33533105 PMC8013687

[13] M. A. Tuncer , H. Sadeghian , M. Sheikhvatan , and M. Toulany , “Causality Association Between COVID‐19 Infection and Aortic Dissection,” Anatolian Journal of Cardiology 26 (2022): 338–339, 10.5152/AnatolJCardiol.2021.1321. 35435848 PMC9366369

[14] S. Mamishi , A. Navaeian , and R. Shabanian , “Acute Aortic Dissection in a Patient With Williams Syndrome Infected by COVID‐19,” Cardiology in the Young 31 (2021): 132–134, 10.1017/S1047951120003236. 33040742 PMC7550877

[15] A. Ramandi , M. A. Akbarzadeh , I. Khaheshi , and M. R. Khalilian , “Aortic Dissection and Covid‐19; A Comprehensive Systematic Review,” Current Problems in Cardiology 48 (2023): 101129, 10.1016/j.cpcardiol.2022.101129. 35139402 PMC8817949

[16] Y. A.‐O. Manla , G. Bhatnagar , N. Khan , et al., “Management of Acute Aortic Services During the COVID‐19 Pandemic: A Retrospective Cohort Study From the Middle East,” Annals of Medicine and Surgery 85, no. 7 (2012): 3279–3283. 10.1097/MS9.0000000000000813 PMC10328633 37427187

